# Case Report: Penile Strangulation Secondary to Hair Tourniquet

**DOI:** 10.3389/fped.2020.00477

**Published:** 2020-08-21

**Authors:** William F. Rawls, Jeffrey T. White, Ahmad Mohamed, Dennis Peppas, Eran Rosenberg

**Affiliations:** ^1^Department of Urology, University of Louisville, Louisville, KY, United States; ^2^Department of Pediatric Urology, Norton Healthcare, Louisville, KY, United States

**Keywords:** penile strangulation, hair tourniquet, penile edema, excessive crying, pediatric emergency

## Abstract

Penile strangulation is a rare condition in children caused by circumferential constriction of the coronal sulcus by constricting material, commonly thin maternal hair. Vague presenting symptoms often makes diagnosis difficult, but delay in diagnosis can lead to a variety of severe complications including urethral injury and penile necrosis. Providers must have a high index of suspicion and carry out a careful examination to identify maternal hair strands that may bury deep within penile edema. We describe two cases of penile strangulation secondary to maternal hair strands that were successfully treated with thorough examination and division of the constricting hair in the emergency department. In both cases, presentation involved penile swelling and erythema which was noticed by caregivers. Once the diagnosis has been made, urgent treatment using depilatory cream or mechanical removal must occur, with urgent referral to specialists if unable to remove to constricting material. Caregivers must also be counseled on appropriate steps to prevent penile hair tourniquet syndrome. Devastating complications can be avoided by early recognition and proper management of the syndrome, but providers must have knowledge of the condition and a high index of suspicion.

## Introduction

Penile strangulation is a rare condition in children caused by entanglement of the coronal sulcus by constricting material. The only sign of penile strangulation may be infant irritability associated with penile edema and may not be recognized or may be misinterpreted for other more common conditions. Delay in diagnosis has potentially devastating consequences.

The most common culprit—strands of thin maternal hair—has great tensile strength and contracts as it dries. Circumferential constriction causes obstruction of venous and lymphatic drainage. Progressive edema reduces arterial blood supply, potentially resulting in necrosis and autoamputation if not promptly treated (1). Direct injury to structures may also occur—the corpora and urethra are especially prone to transection due to thin protective tissue layers (2).

The phenomenon occurs almost exclusively in circumcised males, although cases of hair tourniquets of clitoris, labia, and digits also occur (3, 4). Typical age presentation overlaps with maternal postpartum hair loss, a phenomenon known as telogen effluvium, which affects over 90% of women in the postpartum period (5, 6).

## Case Descriptions

### Case 1

A 2-year old circumcised male presented to the emergency department after being evaluated at his pediatrician's office. Chief complaint at the pediatrician's office was diarrhea and penile rash believed to be due to the diarrhea. The mother first noticed redness and swelling of the glans penis while giving the patient a bath, although she noted that the patient has been grabbing at his penis for the last 3–4 days. A hair was noted upon examination by the pediatrician, but was unable to be successfully removed. Patient was voiding with a normal amount of wet diapers.

**Figure 1:**
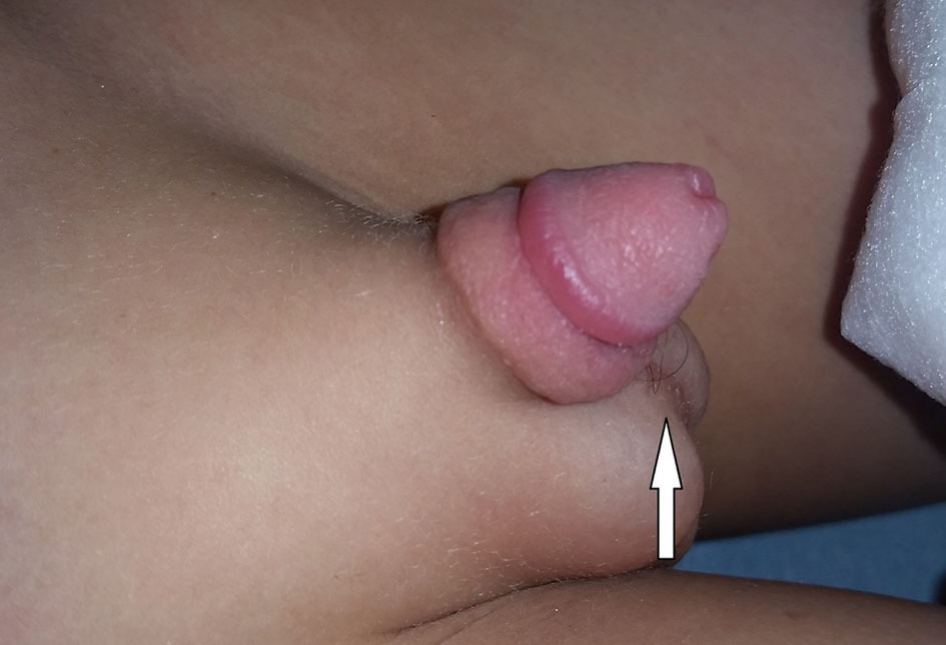
Hair tourniquet with penile edema.

**Figure 2:**
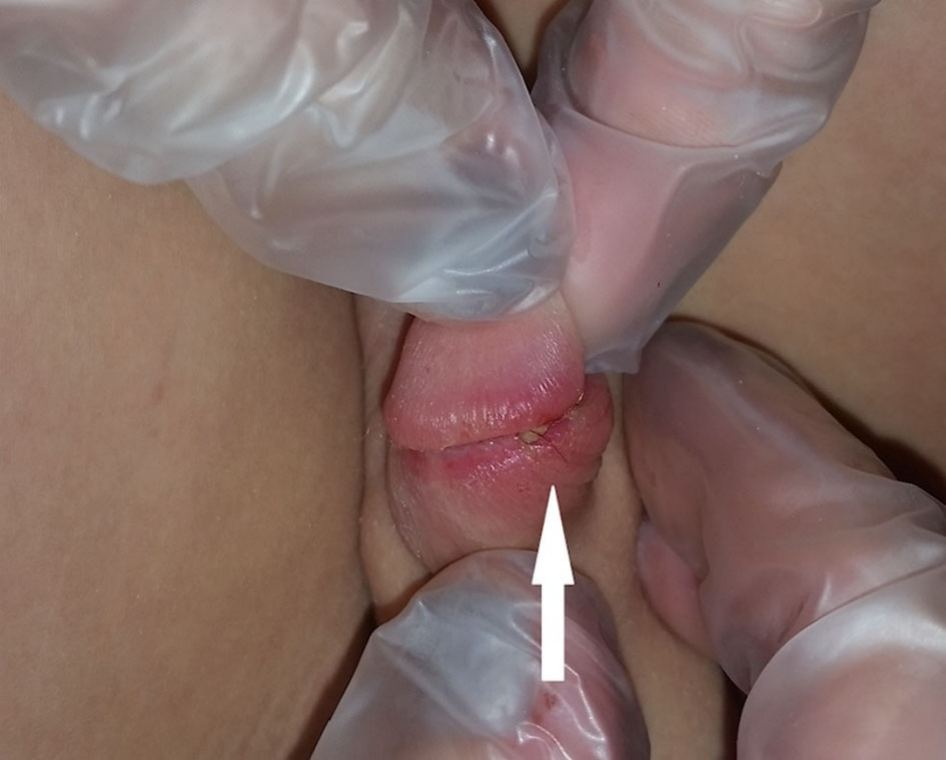
Laceration.

Upon examination, a 2–3 mm circumferential laceration just proximal to the corona was associated with a thin hair tourniquet (Figures 1, 2). The laceration was superficial and did not involve the deep fascia of the penis. The glans was viable with appropriate capillary refill. The hair was successfully removed with scissors in the emergency department and required no sedation. Urinalysis revealed microscopic hematuria; a urethral catheter was placed with no resistance and return of clear yellow urine. Wound care included application of Silvadene cream to the laceration. Upon follow up 7 days later, the catheter was removed and patient was treated for culture-positive urinary tract infection. The laceration was well healed with secondary intention at 1 month follow up.

### Case 2

A 17-month old male with history of trisomy 21 initially presented to their pediatrician with a complaint of penile swelling and irritation for an estimated 12 h. The mother first noticed glanular erythema and swelling after a morning diaper change. He was voiding without difficulty and with normal number of wet diapers. Patient was calm and playful in the office. The pediatrician identified the hair tourniquet, but was unable to remove the hair without proper tools. The patient was promptly transferred to the pediatric urology office.

Physical examination revealed a circumcised male with swelling of the distal shaft and glans. Under the coronal sulcus, there was a circumferential indentation and a hair tourniquet was visualized. Topical anesthetic was applied, then forceps were used to successfully remove multiple hair strands. The procedure was well-tolerated. The patient was observed and discharged home with instructions to apply bacitracin to the affected area. At 1 month follow up with the pediatrician, the patient had a normal genital examination and no genitourinary complaints.

## Discussion

Although penile strangulation secondary to hair tourniquet is rare, it is likely underreported. Less than 100 cases of penile hair strangulation are published in the literature (7). Many clinicians remain unfamiliar with proper recognition and management. Presenting symptoms are often vague: infant irritability, excessive crying, or grabbing at the genitalia may be the only indication that an ischemic process is ongoing (1). Differential diagnoses include infection, insect bites, dermatitis, trauma or congenital constriction bands.

Circumcision is considered a major risk factor for the development of penile hair strangulation (8). Post-circumcision anatomy bares the coronal sulcus. The coronal sulcus serves as a valley for hair to become entrapped and stay well hidden, as was demonstrated by both cases at our institution.

Delay in presentation allows thin hair fibers to become deeply embedded within edematous tissue. Neo-epithelialization over the constricting item may inhibit identification by even the most careful examiner (1). Lighting and a hand-held magnifying glass or loupes are vital tools for proper diagnosis. Probing should be attempted at the lateral aspects of the penis, particularly at any depression (hour-glass deformity) or sharp demarcations. A metal probe with a blunt narrow tip is useful to isolate hair strands. Once the hair is retracted away from tissue, scissors can be used to cut the strands. Providers must consider that numerous hair strands may be involved. Urgent referral to specialists for examination under anesthesia is warranted if mechanical removal is not easily achieved.

Depilatory cream, with the active ingredient calcium thioglycolate, can be applied to the affected extremity and then removed by gentle washing after several minutes (9). Application of depilatory cream has been successful in 64% of cases, some needing a second application of the cream (10). Contraindications to depilatory cream include application to mucosal surfaces; depilation creams will not treat strangulation with other materials such as threads or fabric (5). Application of depilatory cream is a well-tolerated first step in management for primary providers to attempt, although urgent urologic consultation is advised if all hair strands are not clearly removed.

Complications range from penile edema to penile amputation. Badawy et al. described 25 children with complete or partial urethral transection that all required surgical one-stage reconstruction. Four of the 25 had further complications: 2 small urethrocutaneous fistulas and 2 urethral strictures (2). The two anastomotic urethral strictures were successfully managed with endoscopic direct vision internal urethrotomy. The two urethrocutaneous fistulas required re-operation at 1 year with direct closure. The phenomenon has also been associated with one case of hypospadias repair failure. Jesus et al. described a 10-year old boy with a large recurrent distal urethral fistula following penoscrotal hypospadias repair associated with a hair tourniquet at the urethral plate (7).

Though usually an accidental occurrence, some social circumstances warrant further investigation. In some situations, child abuse must be considered. Additionally some young boys intentionally tie thread around the penis to control enuresis (6). Education and prevention strategies are pivotal. Parents should be counseled to wash mother and infant's clothes separately, launder infant clothing inside-out, carefully examine digits and genitals when bathing or changing diapers, frequently brush and dispose of shed hair, and promptly remove any suspect hair strands (6).

Hair tourniquet presentation can mimic other conditions, making diagnosis difficult. Providers must have a high index of suspicion for this serious condition affecting mostly circumcised boys. Early recognition and urgent urologic consultation can prevent serious complications.

## Data Availability Statement

The raw data supporting the conclusions of this article will be made available by the authors, without undue reservation.

## Ethics Statement

Ethical review and approval was not required for the study on human participants in accordance with the local legislation and institutional requirements. The patients/participants provided their written informed consent to participate in this study. Written informed consent was obtained from the minor(s)' legal guardian/next of kin for the publication of any potentially identifiable images or data included in this article.

## Author Contributions

All authors listed have made a substantial, direct and intellectual contribution to the work, and approved it for publication.

## Conflict of Interest

The authors declare that the research was conducted in the absence of any commercial or financial relationships that could be construed as a potential conflict of interest.
